# Acute Effects of Triathlon Race on Oxidative Stress Biomarkers

**DOI:** 10.1155/2020/3062807

**Published:** 2020-01-17

**Authors:** Simona Mrakic-Sposta, Maristella Gussoni, Alessandra Vezzoli, Cinzia Dellanoce, Mario Comassi, Guido Giardini, Rosa Maria Bruno, Michela Montorsi, Anca Corciu, Fulvia Greco, Lorenza Pratali

**Affiliations:** ^1^Institute of Clinical Physiology, National Council of Research (IFC-CNR), ASST Grande Ospedale Metropolitano Niguarda, Milan, Italy; ^2^Institute of Science and Chemical Technology, National Council of Research (SCITEC-CNR), Milan, Italy; ^3^Institute of Clinical Physiology, National Council of Research (IFC-CNR), Pisa, Italy; ^4^Neurology and Neurophysiology Department, Mountain Medicine Center Valle d' Aosta Regional Hospital Umberto Parini, Aosta, Italy; ^5^Department of Clinical and Experimental Medicine, University of Pisa, Italy; ^6^Department of Human Sciences and Promotion of the Quality of Life, San Raffaele Roma Open University, Milan, Italy

## Abstract

The response to strenuous exercise was investigated by reactive oxygen species (ROS) production, oxidative damage, thiol redox status, and inflammation assessments in 32 enrolled triathlon athletes (41.9 ± 7.9 yrs) during Ironman® (IR), or half Ironman® (HIR) competition. In biological samples, inflammatory cytokines, aminothiols (glutathione (GSH), homocysteine (Hcy), cysteine (Cys), and cysteinylglycine (CysGly)), creatinine and neopterin, oxidative stress (OxS) biomarkers (protein carbonyl (PC), thiobarbituric acid-reactive substances (TBARS)), and ROS were assessed. Thirteen HIR and fourteen IR athletes finished the race. Postrace, ROS (HIR +20%; IR +28%; *p* < 0.0001), TBARS (HIR +57%; IR +101%), PC (HIR +101%; IR +130%) and urinary neopterin (HIR +19%, IR +27%) significantly (range *p* < 0.05-0.0001) increased. Moreover, HIR showed an increase in total Cys +28%, while IR showed total aminothiols, Cys, Hcy, CysGly, and GSH increase by +48, +30, +58, and +158%, respectively (range *p* < 0.05-0.0001). ROS production was significantly correlated with TBARS and PC (*R*^2^ = 0.38 and *R*^2^ = 0.40; *p* < 0.0001) and aminothiols levels (range *R*^2^ = 0.17-0.47; range *p* < 0.01-0.0001). In particular, ROS was directly correlated with the athletes' age (*R*^2^ = 0.19; *p* < 0.05), with ultraendurance years of training (*R*^2^ = 0.18; *p* < 0.05) and the days/week training activity (*R*^2^ = 0.16; *p* < 0.05). Finally, the days/week training activity (hours/in the last 2 weeks) was found inversely correlated with the IL-6 postrace (*R*^2^ = ‐0.21; *p* < 0.01). A strenuous performance, the Ironman® distance triathlon competition, alters the oxidant/antioxidant balance through a great OxS response that is directly correlated to the inflammatory parameters; furthermore, the obtained data suggest that an appropriate training time has to be selected in order to achieve the lowest ROS production and IL-6 concentration at the same time.

## 1. Introduction

Triathlon is a multiple-stage competition involving the completion of three continuous and sequential endurance disciplines [1]. Even if many variations of the sport exist, triathlon, in its most popular form, involves swimming, cycling, and running in immediate succession over various distances [2]. Triathlon thereby provides a great opportunity to investigate the effects of multidisciplinary exercises on health, and to this aim, much remains to be done [3]. Long competitions require great power endurance possibly leading to heat stress and dehydration [4, 5], muscle injury [6], oxidative stress [7, 8], inflammation [9, 10], immunologic alterations [11, 12], and cardiac remodeling [13].

Aerobic exercises, particularly ultraendurance exercise, increase the oxygen consumption rate, which, in turn, increases the high reactive oxygen species (ROS) production [14, 15] so antioxidant defenses are mostly required to protect cells from oxidative damage. Overproduction of ROS, potentially damaging cells, particularly macromolecules (i.e. lipids, proteins, carbohydrates, and nucleic acids) [16, 17] in both skeletal muscle and blood [18], is known as oxidative stress (OxS). As widely reported, OxS may result in an inflammatory response by the immune system to protect tissues [12, 19, 20]. Nonetheless, at appropriate concentration, ROS are known to act as important signaling molecules [21, 22].

ROS measurement is difficult because they are produced in low quantities, characterized by extremely short half-life (i.e., t_1/2_: superoxide [O_2_^·‐^] = 10^−4^ s; nitric oxide [NO^·^] = 0.4 s at ambient temperature) and rapid interaction with antioxidants, like thiol residues, and cellular components, molecular oxygen, and metalloproteins. Numerous assays and biomarkers have been used to measure oxidative stress [16, 17]. However, none of them is entirely satisfactory and/or they have been inappropriately used [23].

Electron paramagnetic resonance (EPR) is the only technique capable of providing a direct detection of the “instantaneous” presence of free radical species in a sample [21, 24] and at the same time giving an “intrinsically quantitative” information by adopting a microinvasive measuring method [25]. Indeed, as proportional to the number of the excited electron spins, EPR signal areas can return absolute concentrations once that a stable radical compound is adopted as an internal reference.

Aminothiols are a major species of nonenzymatic antioxidant compounds characterized by reduced sulfhydryl moieties able to directly quench ROS. The specimens are present in all fluids and tissues, furthermore playing important roles in cell signaling, metabolism, and detoxification, and their concentration is associated to OxS [26]. Various aminothiols (i.e., homocysteine (Hcy), cysteine (Cys), cysteinylglycine (CysGly)) are metabolically strictly connected and can be considered the principal interface between changing redox environment and protein activity [8]. Reduced glutathione (GSH) results in the most abundant presence of aminothiols in tissues, and it serves multiple functions to protect from oxidative damage by keeping the intracellular environment in a reduced state [27].

Several studies have investigated alterations of the biochemical responses in athletes involved in ultraendurance exercise, focusing the interest on exercise-induced muscle damage accompanied by the presence of inflammatory mediators and how their modulation can be affected by the intensity, mode, and duration of the exercise challenge [28–30].

Some of these have in particular determined the cytokine responses to ultraendurance exercise, where the immune response can be affected by ROS [30] that are, in turn, able to modulate the acute phase of inflammatory responses as well [31].

Another marker providing information about immune activation is urinary neopterin [32, 33]. Neopterin is an unconjugated pteridine, secreted in large quantities by activated macrophages: it can be used as a clinical marker of activated cellular immunity [33].

In order to clarify the physiological mechanisms involved when the human body is pushed acutely to its limits, a group of athletes performing in the Ironman® triathlon distance competition (IR) and half distance Ironman® triathlon (HIR) were monitored in the present study. In particular, as redox physiology plays an important role in the performance, especially under endurance exercise, redox status modifications in the time course of the competition were investigated by measuring ROS production levels, lipids, and protein OxS damage biomarkers (TBARS and PC), and thiols' redox status in both erythrocytes and plasma. Finally, to complete the picture, inflammatory markers (IL-6, IL-10, IL 1*β*, TNF-*α*, Irisin, and Monocyte Chemoattractant Protein-1), biochemical-hematological profile, and renal function markers (creatinine and neopterin) were assessed. Anthropometrical and training load characteristics influences were analyzed too.

## 2. Materials and Methods

### 2.1. Snapshot of the Race

**Figure 1:**
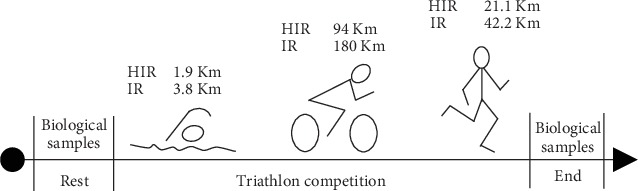
Experimental protocol adopted to measure the OxS parameters on the biological samples collected from the Elbaman (IR) and Elbaman 73 (HIR) selected participants.

The study was performed during the “Elbaman®” race at Elba Island (Italy). Two types of competition were proposed by the organizers (http://www.elbaman.it): “Elbaman” (IR): 3.8 km swim, followed by 180 km bike and 42.2 km run, and at the same time, a competition on half the distance, the Elbaman73 (HIR): 1.9 km swim, followed by 94 km bike and 21.1 km run. A sketch of the experimental protocol and of the race is displayed in Figure 1. On the day of the race, ambient temperature was 23.2°C. During the competition, athletes were allowed to eat and to drink ad libitum what was provided by the organizers.

### 2.2. Subjects

Thirteen HIR (age 42.9 ± 8.8 yrs; height 174.3 ± 5.6 cm) and nineteen IR (age 41.3 ± 7.1 yrs; height 174.9 ± 4.6 cm) athletes were recruited on a volunteer basis among male participants in the competitions. The exclusion criteria were as follows: age < 18 yrs; presence of chronic disease such as hypertension, hypercholesterolemia, and diabetes; use of any medication, antioxidant, or related supplements in the 7 days preceding the study; and unwillingness to sign informed consent. The anthropometric parameters, body mass index (BMI), body fat (FM), free fat masses (FFM), and total body water (TBW) were determined by bipolar bio-impedentiometry (TBF-300A Body Composition Analyzer; Tanita Corporation, Arlington Heights, IL, USA). Before and immediately after the race, blood pressure (BP) was measured by a standard cuff sphygmomanometer, finger O_2_ saturation (SaO_2_) and heart rate (HR) (HR and SaO_2_: by Oximetry-Ohmeda TuffSat, GE Healthcare, Helsinki, Finland) were measured, and the mean value of three evaluations for each parameter was obtained.

The triathletes enrolled were asked to not perform any form of exercise 24 h preceding the race.

Each of the enrolled athletes, with training history and training load for the last two weeks before the race, was administered.

The experimental protocol was approved by the Valle d'Aosta Region Ethical Committee (n. 65943), and all volunteers signed an informed consent according to the Declaration of Helsinki.

### 2.3. Blood and Urine Sample Collection

Athletes underwent two test sessions for venous, capillary blood, and urine sample collections: the first (PRE) was performed 1 day before, while the second (POST) immediately after the end of the race. Approximately 15 mL of blood was drawn from an antecubital vein and collected in heparinized (5 mL) and EDTA- (10 mL) treated vacutainer tubes (Becton Dickinson and Company, UK). Plasma was separated by centrifugation (5702R, Eppendorf, Germany) at 3000 g for 5 min at 4°C. Plasma and erythrocytes samples were immediately stored in multiple aliquots at −80°C until being assayed. Urine samples were also collected both PRE and POST, and aliquots were stored at −80°C until analyses. Samples were thawed only once for analyses, which were performed within two weeks from collection. 50 *μ*L of capillary blood was taken from the fingertip PRE and POST for EPR measurements.

#### 2.3.1. Blood Measurements

*(1) Hematological Parameters*. Hematological parameters (i.e., cholesterol, osmolality, uric acid, and main types of the white blood cells (WBC)) were analyzed by standard methods; inflammatory biomarkers (i.e., TNF-*α*, IL-1*β*, IL-6, IL-10, Monocyte Chemoattractant Protein-1 (MCP-1), and Irisin) were assessed by enzyme-linked immune assays according to the manufacturer's instructions. Detailed procedures and the assessed values for each parameter above reported have been previously reported by some of us [34].

*(2) Oxidative stress (OxS)*.

- ROS Detection. ROS production rate was assessed using the methods of Mrakic-Sposta et al. [7, 25, 35, 67] with an X-band EPR instrument (E-Scan-Bruker BioSpin, GmbH, MA USA), using 50 *μ*L of capillary blood withdrawn from the fingertip and treated immediately with probe solution CMH (1-hydroxy-3-methoxycarbonyl-2,2,5,5-tetramethylpyrrolidine). All samples were stabilized at 37°C by the Temperature and Gas Controller “Bio III” unit, interfaced to the spectrometer. All spectra were collected by acquisition parameters previously reported [7, 25, 35, 67], and Bruker software (Win EPR System, V. 2.11) was adopted for the analysis.
- Thiols Assessment. Total and reduced aminothiols were measured in the erythrocytes and plasma samples according to previously validated methods [36]. Briefly, Tris-(2-carboxyethyl)-phosphine hydrochloride (TCEP) and 4-fluoro-7-sulfamoylbenzofurazan (ABD-F) were used as reducing and derivatizing agents, respectively; reduced aminothiols were assessed by mixing erythrocytes and plasma with 10% trichloroacetic acid (1 : 1 (*v*/*v*)). 100 *μ*L of each supernatant obtained was added with 10 *μ*L of 0.4 M NaOH, 70 *μ*L of 1 M borate buffer pH 11 containing 4 mM EDTA, 30 *μ*L of 1 M borate buffer pH 9.5 containing 4 mM EDTA, and 10 *μ*L of 10 g·L^−1^ ABD-F in borate buffer (1 M, pH 9.5 containing 4 mM EDTA). Samples were incubated at 4°C for 90 min, and then 10 *μ*L was injected into the High-Performance Liquid Chromatography (HPLC) system for analysis. Thiols' separation was performed at room temperature by isocratic HPLC analysis on a Discovery C-18 column (250 × 4.6 mm ID, Supelco, Sigma-Aldrich), eluted with a solution of 0.1 mol·L^−1^ acetate buffer, pH 4.0: methanol, 81 : 19 (*v*/*v*), at a flow rate of 1 mL·min^−1^. Fluorescence intensities were measured by using a fluorescence spectrophotometer (JASCO, Japan, excitation at 390 nm and emission at 510 nm). A standard calibration curve was used to assay sample concentration. The concentration of the oxidized forms was calculated as the difference between the total and the reduced forms
- Protein Carbonyls (PC). The accumulation of oxidized proteins was measured by the reactive carbonyl content. A protein carbonyl (PC) assay kit (Cayman Chemical, U.S.) was used to colorimetrically evaluate the oxidized proteins at 370 nm. The obtained values were normalized to the total protein concentration in a final pellet (absorbance reading at 280 nm), in order to consider the protein loss during the washing steps, as suggested by the kit's user manual
- Thiobarbituric Acid-Reactive Substances (TBARS). Thiobarbituric acid-reactive substance (TBARS) measurement was adopted to detect lipid peroxidation. The used TBARS assay kit (Cayman Chemical, U.S.) allows a rapid photometric thiobarbituric acid malondialdehyde (TBAMDA) adduct detection at 532 nm. A linear calibration curve was computed from pure malondialdehyde containing solutions

#### 2.3.2. Urine Marker Measurements

As the collection of the 24 h urine is not possible, urinary parameters are standardized basing on the amount of the excreted creatinine, since, as well known, in a human subject and in the absence of renal disease, creatinine excretion rate keeps relatively constant.

*(3) Creatinine and Neopterin*. Creatinine and neopterin concentrations were measured by an isocratic high-pressure liquid chromatography (HPLC) method. Briefly, urine samples were thawed and centrifuged at 13000 rpm for 5 min at 4°C; the supernatant was then adequately diluted with the chromatographic mobile phase (15 mM of K_2_HPO_4_, pH 3.0). Neopterin and creatinine levels were measured using a Varian pump (240, autosampler ProStar 410) coupled to a fluorometric detector (JASCO FP-1520, *λ*_ex_ = 355 nm and *λ*_em_ = 450 nm) for neopterin and to a UV-VIS detector (Shimadzu SPD 10-AV, *λ* = 240 nm) for creatinine determinations. Neopterin and creatinine separations were performed at 50°C on a 5 *μ*m Discovery C18 analytical column (250 × 4.6 mm ID, Supelco, Sigma-Aldrich) at a flow rate of 0.9 mL·min^−1^. Linear calibration curves were found over a concentration of 0.125^−1^ mmol·L^−1^ and 1.25^−10^ mmol·L^−1^ for neopterin and creatinine (the score in *μ*mol·L^−1^ was divided by 88.4 to obtain mg·dL^−1^ creatinine), respectively. Inter-assay and intra-assay variation coefficients resulted in less than the 5%.

### 2.4. Statistical Analysis

Data were reported as the mean ± SD. Statistical analysis was performed by using the GraphPad Prism package (GraphPad Prism 8, Software Inc., San Diego, CA). The normality of the data distribution was tested with Shapiro-Wilk's test. The data were normally distributed. Experimental data were compared using ANOVA repeated measures with a Bonferroni posthoc test. Pearson's product moment correlation coefficient (*R*) with 90% confidence intervals (CI) was used to determine the relationships between selected parameters. Prospective power calculation to determine the significant subject number was made by using the Freeware G∗Power software (http://www.psycho.uni-duesseldorf.de/abteilungen/aap/gpower3/). At a power of 80%, a significant population of 11 subjects was calculated, well below the subject's population recruited for the study.

## 3. Results

### 3.1. Anthropometric, Physiological, and Biochemical-Hematological Parameters

Among 32 enrolled athletes, 27 completed the competition. Anthropometric and physiological parameters are reported in Table 1. At POST, weight, FFM, and TBW were found to be decreased in both the IR and HIR groups (of about the 3%). As expected, heart rate was significantly increased immediately after the end of the race: IR: +37% and HIR: +41%.

White blood cell (WBC) count and the percentages of the five types of WBCs in overall count recorded at PRE and POST race are shown in Table 2. Interleukins (IL-1*β*; IL-6, IL-10, and TNF-*α*) and cytokine (MCP-1) levels were also reported.

### 3.2. ROS Production, Oxidative Damage, and Aminothiol Measurements

**Figure 2:**
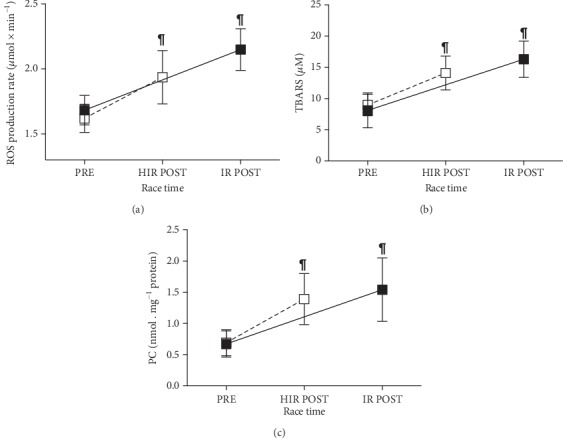
Plots of the (a) ROS production rate (*μ*mol·min^−1^); (b) TBARS (*μ*M), and (c) PC (nmol·mg^−1^ protein) data collected from the HIR (empty square) and IR (full square) groups. Data are expressed as the mean ± SD. The lines connecting PRE and POST data are displayed as guide for the eyes. Changes over time (POST vs. PRE) were significant at ^¶^*p* < 0.0001.

ROS production rate measured in capillary blood and plasmatic OxS biomarkers concentration data is shown in the plot panels of Figure 2. No significant differences in the ROS production rate and oxidative damage biomarkers (TBARS and PC) at basal levels were found between the two (HIR and IR) groups.

The ROS production significantly increased at POST vs. PRE (p < 0.0001) in HIR (1.93 ± 0.21 vs. 1.62 ± 0.10 *μ*mol·min^−1^) and IR (2.15 ± 0.16 vs. 1.68 ± 0.11 *μ*mol·min^−1^) athletes (Figure 2(a)), with significant differences in POST between HIR and IR.

A similar trend POST vs. PRE observation for oxidative damage biomarkers was observed for TBARS in HIR: 14.09 ± 2.70 vs. 8.91 ± 1.72 *μ*M, and in IR: 16.31 ± 2.90 vs. 8.09 ± 2.80 *μ*M (*p* < 0.0001) (Figure 2(b)); PC in HIR: 1.39 ± 0.41 vs. 0.69 ± 0.21, and in IR: 1.54 ± 0.51 vs. 0.68 ± 0.21 nmol·mg^−1^ protein (*p* < 0.0001) (Figure 2(c)).

Aminothiol concentration data measured at PRE and POST sessions from HIR and IR subjects on both plasma and erythrocytes samples are reported in Table 3. At PRE, no significant differences were observed between the two athletes' groups. In plasma, significant (*p* < 0.001) changes at POST vs. PRE, in both the HIR and IR in total and oxidized Cys, were calculated. Furthermore, in IR at POST total Hcy (*p* < 0.05), total and oxidized CysGly (*p* < 0.001), and total and oxidized GSH (*p* < 0.0001), concentrations were found to be significantly increased.

In red blood cells (RBCs), total and reduced GSH concentration data resulted in a significant (*p* < 0.0001) increase in POST vs. PRE, in both the athletes' groups.

### 3.3. Neopterin

**Figure 3:**
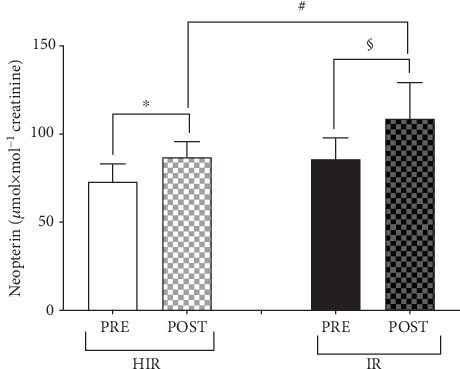
Neopterin concentrations (*μ*mol·mol^−1^ creatinine) measured from the HIR (empty and grey bars) and IR (full bars) athletes. Results are expressed as the mean ± SD. Significantly different data are displayed between brackets. Changes over time (POST vs. PRE) were significant at ^∗^*p* < 0.05, ^#^*p* < 0.01, and ^§^*p* < 0.001.

The renal functional biomarker neopterin data collected on both the HIR and IR athletes' groups at PRE and POST measurement sessions are shown in Figure 3. No significant differences were found at PRE in the two groups. Neopterin concentration significantly increased (POST vs. PRE) in both HIR: 86.88 ± 8.78 vs. 73.01 ± 10.21 (*p* < 0.05), and IR 108.65 ± 20.56 vs. 85.87 ± 11.89 (*p* < 0.001) *μ*mol·mol^−1^ creatinine. A significant difference was found for neopterin POST HIR and IR as well (*p* < 0.01).

### 3.4. Parameter Correlations

In both the HIR and IR groups, the correlations between OxS biomarker data (i.e., ROS production, TBARS, and PC concentrations) and biochemical-hematological parameters were observed. The calculated correlation coefficients (*R*^2^) and the significance levels are reported in Table 4.

**Figure 4:**
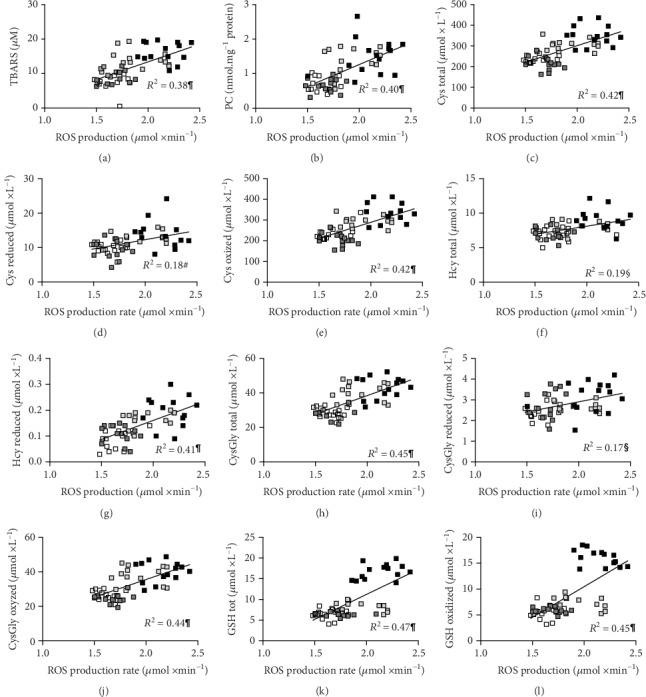
Panel plot showing the individual ROS production rate concentration values (*μ*mol·min^−1^) plotted versus the following: (a) TBARS (*μ*M); (b) PC (nmol·mg^−1^ protein); (c) total, (d) reduced, and (e) oxidized Cys (*μ*mol·L^−1^); (f) total and (g) reduced Hcy (*μ*mol·L^−1^); (h) total, (i) reduced, and (j) oxidized CysGly (*μ*mol·L^−1^); (k) total and (l) oxidized glutathione (*μ*mol·L^−1^) values obtained in HIR athletes at PRE (empty square) and POST (light grey square) and in IR athletes at PRE (dark grey square) and POST (black square). The linear regression lines (solid lines) and the correlation coefficients (*R*^2^) are reported in each panel. Significant relationships: ^#^*p* < 0.01, ^§^*p* < 0.001, and ^¶^*p* < 0.0001.

The linear relationships of the OxS biomarkers (ROS production rate, TBARS, and PC) versus all the other measured parameters' concentration data collected from HIR and IR athletes at PRE and POST session times were calculated. ROS production rate data (Figure 4) were directly correlated to TBARS and PC (*p* < 0.0001, *R*^2^ = 0.38 and *R*^2^ = 0.40) (Figures 4(a) and 4(b)) and aminothiols levels (Figures 4(c)–4(l)).

TBARS concentration data were positively correlated to the following concentrations: total Cys (*p* < 0.0001, *R*^2^ = 0.38), reduced Cys (*p* < 0.01, *R*^2^ = 0.13), and oxidized Cys (*p* < 0.0001, *R*^2^ = 0.38); total Hcy (*p* < 0.001, *R*^2^ = 0.18) and reduced Hcy (*p* < 0.0001, *R*^2^ = 0.32); total CysGly (*p* < 0.0001, *R*^2^ = 0.36) and oxidized CysGly (*p* < 0.001, *R*^2^ = 0.37); and total GSH (*p* < 0.0001, *R*^2^ = 0.46) and oxidized GSH (*p* < 0.0001, *R*^2^ = 0.45).

PC concentration data were positively correlated to the following concentrations: total Cys (*p* < 0.0001, *R*^2^ = 0.30), reduced Cys (*p* < 0.05, *R*^2^ = 0.11), and oxidized Cys (*p* < 0.0001, *R*^2^ = 0.31); total Hcy (*p* < 0.01, *R*^2^ = 0.18) and reduced Hcy (*p* < 0.0001, *R*^2^ = 0.27); total CysGly (*p* < 0.0001, *R*^2^ = 0.21) and oxidized CysGly (*p* < 0.001, *R*^2^ = 0.22); and total GSH (*p* < 0.0001, *R*^2^ = 0.41) and oxidized GSH (*p* < 0.0001, *R*^2^ = 0.39).

Cytokine levels increased (Table 2) and were found correlated to the increase of the correspondent ROS production, lipid peroxidation (TBARS), and protein carbonyl (PC) concentrations. IL-6 and IL-10 were correlated with ROS (*R*^2^ = 0.16 and *R*^2^ = 0.16), TBARS (*R*^2^ = 0.20 and *R*^2^ = 0.17), and PC (*R*^2^ = 0.13 and *R*^2^ = 0.13) stating that these were not significant. Furthermore, Irisin and MCP-1 levels were found correlated with OxS biomarkers: ROS (*R*^2^ = 0.21, *p* < 0.001; *R*^2^ = 0.31, *p* < 0.0001), TBARS (*R*^2^ = 0.13, *p* < 0.01; *R*^2^ = 0.43, *p* < 0.0001), and PC (*R*^2^ = 0.11, *p* < 0.05; *R*^2^ = 0.33, *p* < 0.0001).

**Figure 5:**
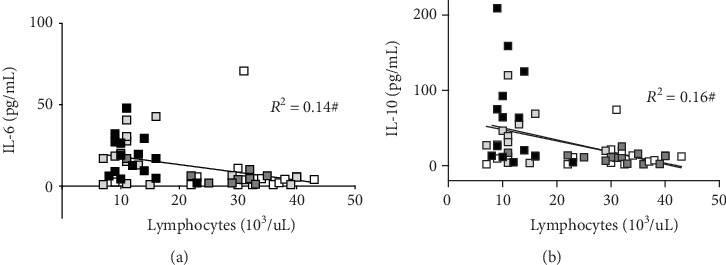
Panel plot showing the lymphocyte count (10^3^/*μ*L) versus cytokine (a) IL-6 and (b) IL-10 concentrations (pg/mL) obtained from HIR subjects at PRE (empty square) and POST (light grey square) and from IR subjects at PRE (dark grey square) and POST (black square). The linear regression lines (solid lines) and the correlation coefficients (*R*^2^) are reported in each panel. Significant relationships: ^#^*p* < 0.01.

Finally, the correlation between lymphocytes and IL-6 and IL-10 cytokine concentration data is displayed in Figures 5(a) and 5(b). In HIR and IR athletes, the levels of cytokines significantly (*p* < 0.01) increased and resulted to be directly correlated with a decrease in circulating lymphocyte concentrations.

Finally, at POST, the individual ROS production rate data was found directly correlated with the athletes' age (*R*^2^ = 0.19, *p* < 0.05), ultraendurance years of training (*R*^2^ = 0.18, *p* < 0.05), and the days/week training activity (*R*^2^ = 0.16, *p* < 0.05). This latter (i.e., training hours spent in two weeks before the race) was found inversely correlated with the POST IL 6 level (*R*^2^ = ‐0.21, *p* < 0.01).

## 4. Discussion

Although up to now the demands of training and competition in an ultra-endurance race are not well described, it has been suggested that triathletes do “extreme amounts of exercises” [3]. Therefore, triathlon Ironman® distance competition can be considered suitable to be studied as a very interesting model of an extreme physiological situation able to promote the deleterious actions of ROS. Most relevant novelty of the present work was the multicomprehensive set of parameters monitored and, in particular, the ROS production rate levels obtained by electron paramagnetic resonance, the only technique able to provide direct and absolute quantitative ROS levels. Oxidative damage biomarkers (TBARS and PC) were monitored too, and for the first time in a triathlon race study, aminothiols redox status in both erythrocytes and plasma was assessed. Moreover, the correlations between the reduced-oxidized forms of various aminothiols (the major nonenzymatic antioxidants in human plasma and erythrocytes), lipid and protein damages, inflammatory response, functional renal impairment, hematological parameters, and training load characteristics influence were calculated.

As widely reported in the literature, during intense aerobic exercise, the rate of oxygen consumption increases due to the significant need for ATP production and this is associated with a large oxygen flux into mitochondria of the working skeletal muscles. Enhances in oxygen consumption are strictly related to ROS production [37]. However, overwhelming ROS production may result in oxidative modifications of lipids, proteins, and/or nucleic acids. In addition, ROS can affect apoptotic processes [38]. OxS, inflammation, and temporary renal function impairment are associated with fatigue and recovery from ultraendurance exercise.

### 4.1. Oxidative Stress Response

OxS represents an imbalance between oxidants, coming from ROS production, and antioxidants in favour of the former ones, leading to a redox signaling and controlling protein disruption [37].

Acute strenuous exercise has been suggested to increase OxS, through the enhanced formation of ROS and nitrogen species [38]. According to these findings, in the present study, despite HIR and IR showing almost similar baseline ROS production rate values, a significant ROS increase was detected at POST: HIR +20% and IR +28%, respectively (see Figure 2(a)). Increased ROS formation after strenuous aerobic exercise includes an inadequate electron transfer through the mitochondrial respiratory chain, inflammatory responses, and heme protein autoxidation [38]. As already pointed out, overwhelming ROS production may result in oxidative lipid and protein modification, although the level of the oxidative damage, leading to alterations in redox homeostasis [39], is also directly related to the exercise type, intensity, and duration [10, 40, 41].

Thus, different kinds of exercise seem to result in different oxidative damage levels, even if data stating the exact influence of the duration of the specific examined exercise on the OxS level has not been ever reported before. Increased levels of various lipid peroxidation indices such as malondialdehyde (MDA), lipid hydroperoxides, F_2_-isoprostane measured after long-distance triathlon, or 50 and 80 km races have been reported in many, but not all, studies found in the literature [8, 38].

In the present study, the completion of an Ironman® distance triathlon (about 13- and 6-hour durations in IR and HIR groups, respectively) led to a significantly higher concentration of lipid peroxidation biomarkers (TBARS: HIR +57%; IR +102%) and protein carbonylation (PC: HIR +101%; IR +126%), when compared to PRE (Figures 2(b) and 2(c)). Indeed, a great ROS amount was generated, and consequently, they may have attacked lipids and proteins [42]. The increased lipid oxidation observed at POST may be related to the increased availability of plasma-free fatty acids, which are needed to support the long competition metabolic requests, as already reported in the literature [43].

On the other hand, protein oxidation can be initiated mainly through hydrogen abstraction from the thiol groups (to generate thiol radicals) or, possibly, from other amino acids such as methionine, as endowed by exposed susceptible functional groups [44].

Thiol groups located on various molecules are known to be central to the redox-sensitive cell signaling mechanisms by means of a reversible process representing a regulated functional switch, so playing a fundamental role in cell biology, biochemistry, and pharmacology [45]. Therefore, simultaneous determination of aminothiols may be a useful tool to study OxS as well as metabolic and redox regulations. The examined aminothiol redox status (see Table 3) demonstrated an increase in plasma and erythrocytes of aminothiol concentrations in both HIR and IR athletes. In particular, plasma-oxidized glutathione concentration exhibited a large increase at POST in HIR (+37%) and IR (171%), when compared to the other examined thiols (i.e., Cys, CysGly, and Hcy) changes.

Previously, Herrmann et al. [46] have hypothesized that exercise duration and intensity are relevant factors in Hcy modulation, while a possible reason for increased Hcy levels after endurance exercise might be the exercise-related hemoconcentration. In agreement with literature data [46], an increase of both total and reduced Hcy levels was found in the present study: HIR +18% and +60%; IR +30% and +90%, respectively, together with a decrease of TBW (-4%) at POST (see Table 1). Therefore, in our opinion and taking into account that both HIR and IR groups exhibited an Hcy increase up to the 90%, hemoconcentration can be regarded as a possible additional factor of influence, as suggested by Herrmann et al. [46]. This is a very important finding in the evaluation of possible detrimental effects of endurance activity, since clinical and epidemiological evidence suggests that plasmatic Hcy levels, although only moderately elevated, are associated with cardiovascular [47, 48] and neurodegenerative diseases [49, 50].

RBCs are extremely vulnerable to oxidative damage during intense extreme exercise, due to the large amount of catalytic iron, and polyunsaturated fatty acid contents that result in highly susceptible to oxidative attack [44]. In fact, the accumulation of primary products of lipid peroxidation and the formation of abnormal protein aggregates are “footprints” of RBC oxidative damage. Moreover, slightly damaged RBCs have been reported to also be susceptible targets for the attack by neutrophils.

The results of the present study showed that, even if a significant perturbation of the aminothiol redox status in plasma was demonstrated, a significant increase in total and reduced GSH was observed in RBC. These results are in line with data previously reported in the literature, where thiol damage resulted in to some extent repair by GSH-mediated reduction [44].

### 4.2. Inflammation Parameters

The intense and strenuous exercise elicited an acute inflammatory response characterized by increasing in the total numbers of circulating neutrophils, monocytes (see Table 2), and OxS level, and in turn, this process stimulated a cytokine production from various cell types and unregulated the inflammatory cascade.

The response to exercise-induced muscle damage typically involves an early invasion of neutrophils that, as reported by the literature, are mobilized by cytokine-mediated demargination from the lung vasculature or released from bone marrow in response to the increased catecholamine levels [51, 52]. Moreover, prolonged fatiguing exercise is accompanied by disturbances in total WBC (i.e., leukocytosis) and leukocyte subset counts (i.e., neutrophilia, monocytosis, and lymphopenia) [53]. Migration of leukocytes during immune surveillance and inflammation is largely determined by their response to chemokines [54].

Excessive strenuous exercise may result in enhanced enteric endotoxin translocation and a cytokine-mediated systemic inflammatory response similar to typical cytokine profile of an acute infectious episode [55, 56].

Our results confirmed that ultraendurance exercise leads to leukocytosis mainly due to an increase in systemic inflammation, as well as neutrophil, and monocytes levels accompanied by a relative decrease of lymphocytes [53].

In agreement with previous studies [20, 57], pro- and anti-inflammatory cytokine elevations: TNF-*α*, IL-1*β*, IL-6, and IL-10 at POST, were observed in the present study, and the results were reported in detail by some of us [34].

Circulating levels of interleukin- (IL-) 6, IL-8, IL-1, receptor antagonist (IL-1ra), and IL-10 have been reported to increase remarkably after endurance exercises longer than 2 h such as marathon [58, 59]. IL-6 is the main cytokine present in the circulation during exercise-contracting skeletal muscle that is the main source of plasma IL-6. Its level was found to increase up to 100-fold, depending on the intensity and duration of the endurance exercise [59].

Leukocytosis following exercise is a well-described stress/inflammatory activation phenomenon in healthy humans: our data showed that leukocytosis after triathlon competition was coincident with the increase of the inflammatory cytokines TNF-*α* and IL-6 [55, 56].

In agreement with literature data [60], lymphocyte count and IL-6, as well as IL-10 cytokines, resulted in being significantly correlated. Moreover, IL-6 and IL-10 levels were found to be correlated to the ROS production rate and oxidative damage of lipids and proteins. In conclusion, all these findings suggested the inflammatory cascade as a related contributor to ROS production during prolonged exercise.

### 4.3. Renal Functionality

Besides the biomarkers of the oxidative stress profile, an increase in systemic inflammation can also be associated to altered biochemical parameters measured in urine and in particular to then level that has previously been suggested to indicate an enhancement of the immune response [61]. Recently, high neopterin production has been associated with immune cellular activation and increased OxS [62]. Therefore, by neopterin assessment, not only the extent of cellular immune activation but also the extent of OxS can be estimated [32].

In the present study, an increase of the urinary neopterin concentration was measured immediately after the race, associated with the OxS rise. These findings suggested that “temporary” renal function impairment could also be regarded as a likely physiological response to a strenuous exercise like ultraendurance competition. Many studies showed that strenuous exercise can be associated with transitory kidney injury although whether this represents a significant insult is not clear [63–65]. Recently, a review on this topic underlined the utility to monitoring kidney function in particular in athletes with acute illness during the race (i.e., any intercurrent illness or gastrointestinal upset), exertional heat stroke, volume depletion, and use of nonsteroidal anti-inflammatory drugs that could be at a higher risk for kidney failure [66]. Further studies are needed to evaluate an optimal biomarker for kidney function assessment after intense exercise in particular in risk subjects.

### 4.4. Parameter Correlations

All data collected in the present study suggested that Ironman triathlon competition elucidated alterations in systemic inflammation and OxS biomarkers both in IR and in HIR. In particular, all changes indicating increased ROS production were found directly related to an increase in the oxidative stress biomarker concentration (Figures 4(a) and 4(b)) and aminothiol levels (Figures 4(c)–4(l)). These results confirm previous observations reporting analogous relationships determined under resting condition [24, 67] or considering the aminothiol delta level between post- and prerunning race [8] also suggesting that the ROS amount generated during triathlon competition modulates the pathways of oxidative damage and at the same time thiol groups are very susceptible to oxidation. Plasma aminothiols (Cys, Hcy, CysGly, and GSH) that are metabolically intercorrelated play an important role in determining the redox status of the cell environment. Thus, an altered plasma aminothiols' profile may reflect a prooxidant status of the athletes at the end of the competition.

The well-known relationship between ROS production and an individual's age [67] can surely be considered a relevant cause of the ROS production rate increase observed according to the average age of ultraendurance experience. In addition, novel findings of the present study are the observed ROS production increase consequent to the days/week training activity and the relationship between the preparation activity load in two weeks before race and the inflammatory marker IL-6 levels recorded from the athletes at POST. Despite the low coefficients calculated (*R*^2^ = 0.16 and 0.21, respectively), the correlation recorded might suggest that the identification of the most appropriate training load for each subject is a crucial issue with the aim to achieve at the same time the lowest ROS production and IL-6 concentration.

## 5. Limitations of the Study

The authors are aware that the present study suffers from certain limitations. First of all, no information on postrace recovery and long-term follow up could be useful to the investigation. On the other hand, it must be pointed out that measurements carried out in the period following the race might be affected by other competitions performed by the athletes so it is actually impossible to evaluate just the single race recovery effects.

Furthermore, during the race, food intake was not standardized so the study lacked of a precise control in water and food intake.

Finally, it would have been interesting to evaluate all the parameters analyzed after each discipline: swimming, cycling, and running to understand possible different behavior in activities, but this is unreliable during a competition.

## 6. Conclusions

Altogether, the entire set of parameters monitored in the present study leads us to conclude that an intense endurance exercise, as the Ironman® distance triathlon competition, causes alterations in the oxidative stress balance (lipid and protein damage, aminothiols), highlighted by the assessed ROS overproduction, changes in inflammatory states (cytokine levels, neopterin concentration), and biochemical hematological parameters. Data indicate that a single competition of ultraendurance exercise is associated with a systemic acute and elevated OxS response with a disturbance in the oxidant/antioxidant balance.

Further studies are needed to shed light about the volume and intensity of exercise which can be considered “healthy” and to investigate possible long-term consequences of repeated and heavy stress as those supported by the triathletes. At the same time, it would be helpful that other biomarkers suitable to increase the specificity between true pathology and physiological adaptation would be identified and to clarify the clinical relevance of such acute changes.

We are all aware that, as recently reported, the endurance and ultraendurance activity is probably the most popular sport worldwide, and it is practiced for recreational, health, and competitive purposes [68]; nonetheless, altogether, the data obtained in the present study put in evidence that an appropriate training time is essential to achieve at the same time the lowest ROS production and IL-6 concentration; in other words, the “right” physical activity will have beneficial effects.

## Figures and Tables

**Table 1:** Anthropometric and physiological parameters from the HIR and IR subjects who ended the race collected at PRE and POST measurement sessions.

	HIR (*n* = 13)			IR (*n* = 14)		
	PRE	POST	*p* value	PRE	POST	*p* value
Weight (kg)	71.2 ± 7.4	69.5 ± 7.7	<0.0001	70.2 ± 5.5	68.2 ± 5.1	0.0002
BMI (kg·m^−2^)	23.4 ± 1.9	22.8 ± 2.0	<0.0001	22.9 ± 1.4	22.3 ± 1.3	ns
FM (kg)	5.3 ± 2.7	5.5 ± 2.4	ns	5.1 ± 2.5	5.5 ± 2.0	ns
FFM (kg)	66.1 ± 5.9	64.1 ± 6.0	<0.0001	65.1 ± 5.6	62.7 ± 5.8	<0.0001
TBW (%)	48.2 ± 4.3	47.0 ± 4.4	<0.0001	47.6 ± 4.1	45.9 ± 3.5	<0.0001
HR (bpm)	60.5 ± 8.7	85.2 ± 17.2	0.0002	59.1 ± 17.4	80.8 ± 14.2	0.0039
SBP (mmHg)	125.5 ± 23.4	141.2 ± 11.5	ns	123.7 ± 16.9	138.4 ± 12.3	ns
DBP (mmHg)	72.5 ± 13.5	81.2 ± 6.4	ns	72.5 ± 10.7	79.5 ± 16.7	ns
SaO2 (%)	97.8 ± 0.6	96.6 ± 1.3	ns	97.6 ± 0.9	97.4 ± 1.5	ns

BMI: body mass index; FM: fat mass; FFM: fat-free mass; TBW: total body water; HR: heart rate; SBP: systolic blood pressure; DBP: diastolic blood pressure; SaO_2_: arterial oxygen saturation (%). Data are reported as the mean ± SD. ns: not significant level.

**Table 2:** White blood cell (WBC) count (10^3^/*μ*L) and inflammatory markers: IL-1*β*, IL-6, IL-10, TNF-*α*, and MCP-1 (pg/mL), determined at PRE and POST sessions from HIR and IR athletes. Data are reported as the mean ± SD. The types of leukocytes, classified in the standard ways, are reported as a percentage (%) of the total WBC together with the significance levels (POST vs. PRE).

	HIR			IR	
	PRE	POST	*p* value	PRE	POST	*p* value
WBC (10^3^/*μ*L)	6.45 ± 3.29	14.27 ± 3.19	<0.05	5.23 ± 0.83	16.85 ± 2.37	<0.05
Leukocytes (%)						
Neutrophils	60	77	<0.0001	58	78	<0.0001
Eosinophils	2.3	3	ns	2.2	2.7	ns
Basophiles	0.4	0.5	ns	0.4	0.5	ns
Lymphocytes	31	11	<0.05	33.5	11.4	<0.05
Monocytes	6.6	8.5	0.0013	5.8	7	<0.0001
^∗^Inflammatory markers (pg/mL)						
IL-1*β*	17.7 ± 16.5	30.2 ± 47.0	<0.05	20.9 ± 17.1	25.3 ± 21.7	ns
IL-6	8.9 ± 18.8	18.9 ± 13.7	<0.05	4.2 ± 2.7	18.9 ± 13.1	<0.05
IL-10	14.2 ± 19.2	35.0 ± 32.8	<0.05	11.5 ± 6.6	63.5 ± 65.9	<0.05
TNF-*α*	19.0 ± 30.0	19.0 ± 18.9	ns	10.9 ± 4.3	12.7 ± 3.1	ns
MCP-1	312 ± 139	852 ± 284	<0.05	337 ± 165	1116 ± 598	<0.05

^∗^Inflammatory marker data were previously reported by some of us [34].

**Table 3:** Aminothiol redox status measured in plasma and red blood cells (RBCs) on HIR and IR athletes before (PRE) and immediately after (POST) the race. Concentrations of the various forms are expressed as *μ*mol·L^−1^.

Plasma	HIR			IR		
	PRE	POST	*p* value	PRE	POST	*p* value
Cysteine						
Total	239.42 ± 17.81	305.36 ± 26.11	<0.0001	213.51 ± 31.28	351.38 ± 50.19	0.0007
Reduced	10.08 ± 1.01	12.79 ± 1.73	ns	9.15 ± 2.77	13.92 ± 4.04	ns
Oxidized	229.34 ± 18.14	292.57 ± 25.99	<0.0001	204.35 ± 30.04	337.45 ± 47.45	0.0006
Homocysteine						
Total	6.95 ± 0.87	8.19 ± 0.76	ns	6.91 ± 0.79	8.99 ± 1.61	0.0147
Reduced	0.10 ± 0.04	0.16 ± 0.03	ns	0.10 ± 0.04	0.19 ± 0.06	ns
Cysteinylglycine						
Total	29.66 ± 3.06	40.90 ± 5.13	ns	27.35 ± 3.00	43.24 ± 6.25	0.0004
Reduced	2.34 ± 0.32	2.71 ± 0.31	ns	2.78 ± 0.68	3.17 ± 0.75	ns
Oxidized	27.31 ± 2.91	38.19 ± 5.18	ns	24.56 ± 2.68	40.07 ± 5.92	0.0004
Glutathione						
Total	6.22 ± 1.16	8.18 ± 1.15	ns	6.47 ± 0.54	16.73 ± 1.86	<0.0001
Reduced	0.92 ± 0.37	0.92 ± 0.39	ns	0.57 ± 0.25	0.70 ± 0.49	ns
Oxidized	5.30 ± 1.31	7.26 ± 1.26	ns	5.90 ± 0.54	16.03 ± 1.59	<0.0001
RBCs						
*Cysteine*						
Total	64.67 ± 8.61	74.69 ± 9.23	ns	62.16 ± 7.67	72.21 ± 8.89	ns
Reduced	10.45 ± 1.45	10.85 ± 1.53	ns	11.13 ± 1.77	11.94 ± 1.79	ns
Oxidized	54.22 ± 8.23	63.84 ± 9.00	ns	51.03 ± 7.07	60.27 ± 8.46	ns
Homocysteine						
Total	2.94 ± 0.39	3.11 ± 0.43	ns	2.61 ± 0.20	3.06 ± 0.29	ns
Reduced	1.17 ± 0.11	1.09 ± 0.13	ns	1.14 ± 0.17	1.19 ± 0.27	ns
Cysteinylglycine						
Total	2.16 ± 0.44	2.51 ± 0.34	ns	2.17 ± 0.41	2.53 ± 0.27	ns
Reduced	0.47 ± 0.37	0.62 ± 0.31	ns	0.56 ± 0.17	0.66 ± 0.16	ns
Oxidized	1.69 ± 0.73	1.89 ± 0.38	ns	1.61 ± 0.46	1.87 ± 0.35	ns
Glutathione						
Total	1691.46 ± 134.29	1957.69 ± 194.03	<0.0001	1870.35 ± 211.98	2160.42 ± 181.61	<0.0001
Reduced	1507.52 ± 141.05	1677.72 ± 168.83	<0.0001	1681.73 ± 244.14	1865.54 ± 219.23	<0.0001
Oxidized	183.93 ± 58.56	279.98 ± 71.24	ns	188.62 ± 57.34	294.88 ± 96.32	ns

**Table 4:** Correlation coefficients (*R*^2^) and *p* significance levels between biochemical-hematological and OxS-selected parameters determined from all the athletes.

Biochemical-hematological parameters						
	ROS	*p* value	TBARS	*p* value	PC	*p* value
Na (mEq/L)	*R* ^2^ = 0.11	0.0142	—	ns	—	ns
Osmolarity (mOsm/L)	*R* ^2^ = 0.33	<0.0001	*R* ^2^ = 0.19	0.012	*R* ^2^ = 0.25	<0.0001
Ferritin (ng/mL)	—	ns	—	ns	—	ns
Transferrin (mg/dL)	—	ns	—	ns	—	ns
Hb (g/dL)	*R* ^2^ = 0.11	0.0132	—	ns	—	ns
MCV (*ϕ*)	—	ns	—	ns	—	ns
Platelets (10^9^/*μ*L)	—	ns	*R* ^2^ = 0.27	<0.0001	*R* ^2^ = 0.17	0.0021
Neutrophils (10^3^/*μ*L)	*R* ^2^ = 0.39	0.0023	*R* ^2^ = 0.61	<0.0001	*R* ^2^ = 0.52	<0.0001
Eosinophils (10^3^/*μ*L)	—	ns	—	ns	—	ns
Lymphocyte (10^3^/*μ*L)	*R* ^2^ = 0.33	<0.0001	*R* ^2^ = 0.50	<0.0001	*R* ^2^ = 0.53	<0.0001
Monocytes (10^3^/*μ*L)	—	ns	—	ns	—	ns
HDL cholesterol (mmol/L)	*R* ^2^ = 0.24	0.0002	*R* ^2^ = 0.12	0.0120	—	ns
Urea (mg/dL)	*R* ^2^ = 0.26	<0.0001	*R* ^2^ = 0.36	<0.0001	*R* ^2^ = 0.42	<0.0001
Uric acid (mg/dL)	*R* ^2^ = 0.36	<0.0001	*R* ^2^ = 0.24	0.0002	*R* ^2^ = 0.28	<0.0001

## Data Availability

The data used to support the findings of this study are included within the article.

## Abbreviations

IR: Ironman®
HIR: Half Ironman®
BMI: Body mass index
SaO_2_: Arterial oxygen saturation
HR: Heart rate
FM: Fat mass
FFM: Fat-free mass
TBW: Total body water
SBP: Systolic blood pressure
DBP: Diastolic blood pressure
OxS: Oxidative stress
EPR: Electron paramagnetic resonance
ROS: Reactive oxygen species
TBARS: Thiobarbituric acid reactive substances
PC: Protein carbonyls
GSH: Glutathione
Hcy: Homocysteine
Cys: Cysteine
CysGly: Cysteinylglycine
TNF-*α*: Tumor necrosis factor-*α*
IL-1*β*, IL-6, IL-10: Interleukin-1*β*, 6, and 10
MCP-1: Monocyte Chemoattractant Protein-1.
